# The impact of genetic polymorphisms on the pharmacokinetics of efavirenz in African children

**DOI:** 10.1111/bcp.12934

**Published:** 2016-04-25

**Authors:** Andrzej Bienczak, Adrian Cook, Lubbe Wiesner, Adeniyi Olagunju, Veronica Mulenga, Cissy Kityo, Addy Kekitiinwa, Andrew Owen, A. Sarah Walker, Diana M. Gibb, Helen McIlleron, David Burger, Paolo Denti

**Affiliations:** ^1^ Division of Clinical Pharmacology, Department of MedicineUniversity of Cape TownCape TownSouth Africa; ^2^ MRC Clinical Trials Unit at University College LondonLondonUK; ^3^ Department of Molecular and Clinical PharmacologyUniversity of LiverpoolLiverpoolUK; ^4^ University Teaching Hospital, Department of Paediatrics and Child HealthLusakaZambia; ^5^ Joint Clinical Research CentreKampalaUganda; ^6^ Baylor College of Medicine Bristol Myers Squibb Children's Clinical Centre of Excellence, Kampala, Uganda / Gulu Regional Centre of ExcellenceGuluUganda; ^7^ Department of PharmacyRadboud University Nijmegen Medical CentreNijmegenthe Netherlands

**Keywords:** Africa, children, *CYP2B6*, efavirenz, pharmacogenetics

## Abstract

**Aims:**

Using a model‐based approach, the efavirenz steady‐state pharmacokinetics in African children is characterized, quantifying demographic and genotypic effects on the drug's disposition. Simulations are also conducted allowing prediction of optimized doses of efavirenz in this population.

**Methods:**

We modelled the steady‐state population pharmacokinetics of efavirenz in Ugandan and Zambian children using nonlinear mixed‐effects modelling. Individual mid‐dose efavirenz concentrations were derived and simulations explored genotype‐based dose optimization strategies.

**Results:**

A two‐compartment model with absorption through transit compartments well described 2086 concentration‐time points in 169 children. The combined effect of single nucleotide polymorphisms (SNPs) 516G>T and 983T>C explained 44.5% and 14.7% of the variability in efavirenz clearance and bioavailability, respectively. The detected frequencies of composite *CYP2B6* genotype were 0.33 for 516GG|983TT, 0.35 for 516GT|983TT, 0.06 for 516GG|983TC, 0.18 for 516TT|983TT, 0.07 516GT|983TC and 0.01 for 516GG|983CC. The corresponding estimated clearance rates were 6.94, 4.90, 3.93, 1.92, 1.36, and 0.74 l h^−1^ for a 15.4 kg child and median (95% CI) observed mid‐dose concentrations 1.55 (0.51–2.94), 2.20 (0.97–4.40), 2.03 (1.19–4.53), 7.55 (2.40–14.74), 7.79 (3.66–24.59) and 18.22 (11.84–22.76) mg l^−1^, respectively. Simulations showed that wild‐type individuals had exposures at the bottom of therapeutic range, while slower metabolizers were overexposed.

**Conclusions:**

Dosage guidelines for African children should take into consideration the combined effect of SNPs *CYP2B6* 516G>T and 983T>C.

## What is Already Known about this Subject

- High variability in efavirenz pharmacokinetics is largely contributed by SNPs in *CYP2B6*: 516G>T and 983T>C.
- SNP 983T>C is virtually absent in individuals of European ancestry.
- No previous studies quantified the effect of 983T>C on efavirenz clearance in children using a model‐based approach or recommended dose optimization strategies accounting for this SNP.

## What this Study Adds

- We propose a model concomitantly accounting for effect of weight and *CYP2B6* 516G>T|983T>C variants in African children.
- Using the model we simulated and compared exposures in weight bands between *CYP2B6* metabolic subgroups and suggested a dose optimization strategy adjusting for effect of both 516G>T and 983T>C.

## Introduction

Efavirenz is a non‐nucleoside reverse transcriptase inhibitor (NNRTI) commonly used in first‐line antiretroviral treatment (ART) for HIV‐1 infected adults and children over 3 years old 1, 2. Due to its ease of dosing (the long half‐life allows once daily administration), proven efficacy, ability to be used with anti‐TB drugs, and availability of cheap generic formulations, it is especially widely used in Africa.

Suboptimal efavirenz exposures have previously been related to treatment failure and high concentrations to central nervous system (CNS) side‐effects 3, 4. Numerous studies reported very large between‐subject variability (BSV) in efavirenz pharmacokinetics (PK) 3, 5, 6, 7. This variability is attributed largely to single nucleotide polymorphisms (SNPs) in the *CYP2B6* gene which encodes the key metabolizing enzyme. The loss‐of‐function polymorphism, 516G>T (rs3745274) 4, 7, 8, 9 alters drug metabolism to the extent that dose adjustment based on *CYP2B6* 516G>T genotype is currently under investigation in children 10, 11. The proportion of slow metabolizers varies among different populations and is relatively high among black Africans 4, 6, 8, 9, 12. In addition, efavirenz concentrations are affected by the functional polymorphisms *CYP2B6* 983T>C (rs28399499) 8, 9, 785A>G (rs2279343) 13, 14 and 15582C>T (rs4803419) 8, 12, which are reported predominantly in black African and African‐American patients; by polymorphisms involving its accessory pathways, including *CYP2A6*, *CYP3A4* and *UGT* 15, 16, 17; and in genes coding nuclear receptors CAR (NR1|3) and PXR (NR1|2), which regulate enzyme expression 18, 19. PK variability has also been linked to several physiological and environmental factors, such as sex 6, 13, 20, ethnicity 6, 21, 22, formulation type 2, 23, concomitant food 2 or co‐medication (e.g. zidovudine 22, rifampicin and isoniazid 7, 22, 24), and adherence 25. However, reports on these effects have been to some extent contradictory and vary between adults and children.

Several investigators have reported a high proportion of subtherapeutic efavirenz concentrations in children, highlighting the need for optimization of paediatric dosing guidelines 26, 27, 28. The aim of this analysis was therefore to characterize the steady‐state PK of efavirenz in the largest cohort of African children reported so far, quantifying demographic and genotypic effects on efavirenz disposition, and thus allowing prediction of optimized doses of efavirenz in this population.

## Methods

PK and other data from two studies in African children from Uganda and Zambia were pooled together: CHAPAS‐3 29 (Children with HIV in Africa – Pharmacokinetics and Adherence/Acceptability of Simple antiretroviral regimens) and ARROW 28, 30 (Anti‐Retroviral Research for Watoto).

## CHAPAS‐3

Efavirenz was dosed once daily, either in the morning or at night, following modified WHO 2010 guidelines (Table 4), using a new paediatric double‐scored 600 mg efavirenz tablet (provided by Cipla Pharmaceuticals, India) that can be split into two or three parts enabling administration of doses of 200, 300, 400 or 600 mg.

All included patients took part in sparse PK sampling on clinic visits at weeks 6, 36, and every 24 weeks thereafter until the end of the study. The self‐reported time of the last dose was recorded. Additional intensive PK sampling was conducted in the first patients enrolled in each WHO weight band at week 6 and in all patients who acquired tuberculosis during the study (4–10 weeks after tuberculosis treatment initiation and again 4–10 weeks after tuberculosis treatment cessation). Children in this intensive PK substudy were advised to take efavirenz in the morning for 6 weeks prior to sampling and the drug intake on the PK day was observed. Plasma was separated and stored at −80°C until transportation on dry ice for drug concentration assay.

Plasma efavirenz concentrations from the intensive PK were assayed using ultra high‐performance liquid chromatography (HPLC) at the Department of Clinical Pharmacy of Radboud University Nijmegen Medical Centre, Nijmegen, the Netherlands. The method produced linear results over the range of 0.0517 to 15.51 mg l^−1^. The lower limit of quantification was 0.05 mg l^−1^. The intraassay and interassay coefficients of variation (CV) were 1.01–5.31% and 0.1–1.63%, respectively. Relative error of the method ranged from 97.7% to 104.5% 31. Plasma efavirenz concentrations from sparse PK were determined by liquid chromatography tandem mass spectrometry (LC–MS/MS) in the Division of Clinical Pharmacology, University of Cape Town, South Africa. The method was accurate over the range of 0.0195–20 mg l^−1^. The lower limit of quantification was 0.0195 mg l^−1^. The interassay CV and residual error (RE) were 3.59–5.78% and −2.57–0.92%, respectively, and the intraassay CV and RE were 1.50–9.67% and −6.00–4.28%, respectively 32.

Both laboratories participate in international quality assurance and proficiency testing schemes and are expected to have comparable standards, although no cross‐validation was performed for the assays. Systematic differences between the labs and assays were tested as covariates in the PK model (see Population pharmacokinetic analysis/Covariates).

## Arrow

Efavirenz was dosed once‐daily, either in the morning or at night, according to modified WHO 2006 paediatric recommendations. The tested formulations included 50, 100 and 200 mg capsules; and half or whole 600 mg tablets (provided by the national ART programme in Uganda).

Children in a PK substudy 28 were sampled on two occasions: 36 and 40 weeks after starting ART. Eligible children were advised to take efavirenz in the morning for 4 weeks prior to sampling and drug intake on the PK day was observed. Samples were stored and assayed at the Department of Clinical Pharmacy of Radboud University Nijmegen Medical Centre, Nijmegen using the same methods as described above.

### Genotyping

Genotyping was performed by allelic discrimination real‐time PCR assay on a DNA Engine Chromo4 system (Bio‐Rad Laboratories, Inc., Hercules, CA, USA). The PCR protocol involved an initial denaturation step at 95°C for 15 min, followed by 50 cycles of amplification at 95°C for 15 s and final annealing at 60°C for 1 min. TaqMan^®^ Genotyping Master Mix and assays for *CYP2B6*‐516G>T (rs3745274, C_7817765_60), *CYP2B6*‐983T>C (rs28399499, C_60732328_20), *CYP2B6*‐c.485‐18C>T (rs4803419; C_7817764_10), *CYP2B6*‐499C>G (rs3826711, C__27522377_10; ARROW patients only), CYP3A4*22 (rs35599367, C__59013445_10), *CYP3A5*‐6986G>A (rs776746, C__59013445_10), *NR1I3* (rs3003596, C__16194070_10; rs2307424, C__25746794_20), *NR1I2*‐63396C>T (rs2472677, C__26079845_10) were obtained from Life Technologies Ltd (Paisley, Renfrewshire, UK). CYP2B6*4 (785A>G, rs2279343) and CYP2B6*29 copy number assay were performed on samples from ARROW study only using previously described custom TaqMan assays 33, 34. Opticon Monitor^®^ version 3.1 (Bio‐Rad Laboratories, Inc., Hercules, CA, USA) was used to obtain allelic discrimination plots and make allele calls. The assays were performed at the Department of Molecular and Clinical Pharmacology, University of Liverpool, Liverpool, UK.

The distribution of the genotypes was evaluated for compliance with Hardy–Weinberg equilibrium using the exact test conducted using R‐package “genetics” 35.

### Population pharmacokinetic analysis

#### Model building

The steady‐state efavirenz PK was analysed using nonlinear mixed‐effects modelling with software NONMEM VII (version 7.3) 36 and the first‐order conditional estimation method with interaction. PsN 4.4.0, Pirana and Xpose were used to facilitate modelling and for model diagnostics 37. The model was developed and validated in accordance with standard methods described in the literature 38. For the structural model, one‐, two‐, and three‐compartment models with first‐order absorption and elimination were tested, as well as time lag or transit‐compartment absorption 39 and hepatic first‐pass model 40. Between‐subject and ‐occasion variability (BSV, BOV) were tested on PK parameters assuming lognormal distribution. Residual unexplained variability (RUV) was tested using a combined proportional and additive error. Data below level of quantification (BLQ) were included in the analysis by imputing half of the lower limit of quantification (LLOQ) of the corresponding assay as suggested in Beal *et al.* (M6 method) 41. Implausible samples and PK profiles were identified using extreme values of CWRESI and their exclusion was evaluated based on visual checks.

Model development and covariate selection was guided by the NONMEM objective function value (OFV), inspection of goodness‐of‐fit (GOF) plots and visual predictive checks (VPCs), biological plausibility and clinical relevance. OFV (proportional to −2 log‐likelihood of the data) was assumed to be χ^2^‐distributed and a drop of 3.84 or more between two hierarchical models after inclusion of one additional parameter (df = 1) was considered a significant improvement (*P* = 0.05). Stability and robustness of the final model, together with precision of its parameter estimates, was evaluated through a nonparametric bootstrap (*n* = 200).

Intensive and sparse data were included in the model development process in a stepwise manner as suggested in Svensson *et al.* 42, starting with intensive PK data from CHAPAS‐3, followed by the intensive data from ARROW, and finally the sparse PK data from CHAPAS‐3.

The model‐derived empirical Bayesian estimates for the individual parameters were used to predict steady‐state mid‐dose concentrations (measures 12 h after dose) for each sampling occasion and patient.

#### Covariates

Allometric scaling was added to the model at an early development stage as previously suggested 43. The effect of maturation of metabolic pathways on PK parameters was tested using post‐menstrual age (gestation‐adjusted age) as a predictor. Both a power function or a sigmoidal model with and without Hill coefficient were tested 43. Besides weight and age, the other covariates tested were: tuberculosis co‐treatment, study site, nucleoside reverse transcriptase inhibitor (NRTI) backbone, sex, weight‐for‐age Z‐score (WAZ) and height‐for‐age Z‐score (HAZ), drug formulation, the effect of splitting tablets used in CHAPAS‐3 (inferred from total daily dose) and genotype information (SNPs listed above). The potential differences between assays and lab procedures for the quantification of drug concentrations were tested in the model as proportionality and correction factors on RUV.

Missing genotype values were imputed using mixture modelling with frequencies fixed to those observed in the rest of the cohort as previously suggested by Keizer *et al.* 44.

#### Simulations

The final model was used to simulate exposures after administration of efavirenz with the formulation given in CHAPAS‐3 and using a dataset of subjects with a uniform distribution of weights ranging from 10 to 40 kg, in 0.1 kg steps (300 individuals simulated 100 times). Several dosing strategies were explored. To avoid generating implausibly extreme values, the maximum variability for each random effect was limited to three standard deviations. Data were analysed and plots generated using R 35.

## Results

### Demographic results and samples

This analysis included data from 128 children from CHAPAS‐3 and 41 children from ARROW. Relevant subject characteristics including the genotype frequencies for the tested SNPs are presented in Table 1. All tested genotypes were in Hardy–Weinberg equilibrium (HWE). For SNPs rs35599367, rs3826711 and CYP2B6*29, all patients were homozygous for the common allele and HWE was not calculated. The genotype information was missing for five children from ARROW and two from CHAPAS‐3, who were assigned by the mixture model as follows: two as 516GG|983TT, four as 516GT|983TT, and one as 516TT|983TT.

**Table 1:** Demographic characteristics

**Characteristics**	**ARROW**	**CHAPAS‐3**		**Combined**
	**iPK**	**iPK**	**sPK**	
**No. of children** *	41	51	128	169
**No. of samples**	611	474	1002	2087
**Sampling schedule**	0 h, 1 h, 2 h, 4 h, 6 h, 8 h, 12 h, 24 h		2 samples 2 h apart	
**No. of samples excluded**	9	8	5	22
**Age (years)** **	7.6 (4.0–12.5)	4.5 (2.1–13.8)		4.7 (2.1–13.8)
**Weight (kg)** **	20.0 (14.0–30.0)	15.0 (7.8–29.9)		15.5 (7.8–30.0)
**Sex (M/F)**	17/24	63/65		80/89
**Race**	Black African			
***CYP2B6* 516GT (rs3745274; HWE *P* = 1)** †				
**GG**	16 (44%)	49 (39%)		65 (40%)
**GT**	14 (39%)	53 (42%)		67 (41%)
**TT**	6 (17%)	24 (19%)		30 (19%)
**MAF**	0.36	0.40		0.39
***CYP2B6* 983T>C (rs28399499; HWE *P* = 0.6)** **†**				
**TT**	33 (92%)	106 (84%)		139 (86%)
**TC**	3 (8%)	19 (15%)		22 (14%)
**CC**	0 (0%)	1 (1%)		1 (1%)
**MAF**	0.04	0.08		0.07
***CYP2B6* 15582C>T (rs4803419; HWE *P* = 1)** **†**				
**CC**	32 (89%)	113 (90%)		145 (90%)
**TC**	4 (11%)	13 (10%)		17 (10%)
**MAF**	0.06	0.05		0.05
CYP3A4*22 (rs35599367) **†**				
**GG**	36 (100%)	126 (100%)		162 (100%)
***CYP3A5* 6986G>A (rs776746; HWE *P* = 0.57)** **†**				
**GG**	1 (3%)	2 (2%)		3 (2%)
**GA**	7 (19%)	41 (33%)		48 (30%)
**AA**	28 (78%)	83 (66%)		111 (69%)
**MAF**	0.12	0.18		0.17
**NR1I3 (rs3003596; HWE *P* = 0.34)** **†**				
**AA**	7 (19%)	30 (24%)		37 (23%)
**AG**	18 (50%)	55 (44%)		73 (45%)
**GG**	11 (31%)	41 (33%)		52 (32%)
**MAF**	0.46	0.46		0.46
**NR1I3 540 C>T (rs2307424; HWE *P* = 1)** **†**				
**TT**	0	1 (1%)		1 (1%)
**CT**	3 (8%)	23 (18%)		26 (16%)
**CC**	33 (92%)	102 (81%)		135 (83%)
**MAF**	0.04	0.10		0.09
**NR1I2 63396C>T (rs2472677; HWE *P* = 0.07)** **†**				
**CC**	13 (36%)	43 (34%)		56 (35%)
**CT**	16 (44%)	72 (57%)		88 (54%)
**TT**	7 (19%)	11 (9%)		18 (11%)
**MAF**	0.42	0.37		0.38
CYP2B6*4 785A>G (rs2279343; HWE *P* = 0.47) ‡				
**AA**	16 (44%)			
**AG**	14 (39%)	not tested		
**GG**	6 (17%)			
**MAF**	0.36			
***CYP2B6* 499C>G (rs3826711)** ‡				
**CC**	36 (100%)	not tested		
CYP2B6*29 ‡				
*1 /*1	36 (100%)	not tested		

Data are median (range) or no. (%) of subjects.

* 51 children in the CHAPAS‐3 study who underwent both intensive and sparse sampling are counted in both categories.

** Baseline values.

† 162 pts from both CHAPAS‐3 and ARROW studies.

‡ 36 pts from ARROW study. HWE, Hardy–Weinberg equilibrium; iPK, intensive sampling; MAF, minor allele frequency; sPK, sparse sampling.

From CHAPAS‐3, 61 intensively sampled PK profiles (a total of 474 samples) and 510 sparse PK profiles (1002 samples, 1–2 per occasion) were available. The PK data were collected from 6 weeks after starting efavirenz up to a maximum of 132 weeks. There were up to 7 PK sampling visits per child. Of 14 children who acquired tuberculosis, nine had at least one intensively sampled PK profile on efavirenz with tuberculosis treatment. The only BLQ measurement from intensive PK substudy was a pre‐dose measurement and all eight samples from that PK visit for that patient were excluded from the analysis as it was deemed likely not to be in steady state due to poor adherence. Within the sparse data, 15 samples were BLQ and were included by imputing half LLOQ, i.e. 0.00975 mg l^−1^. From the ARROW study, 611 intensive PK samples from 82 PK visits (two visits per patient) were available. Data from one visit were discarded due to an implausible PK profile, possibly caused by mismatch of samples. No samples were BLQ.

### Population pharmacokinetics

The data were best described using a two‐compartment model with first‐order elimination and transit compartment absorption 39. Final parameter estimates, their precision (obtained through a bootstrap) and statistical significance for the inclusion of the covariate and random effects (based on drop in OFV) are presented in Table 2. The PK parameters were estimated relative to oral bioavailability whose typical value was fixed to one due to lack of intravenous data. Adequate fit of the model was confirmed by a GOF plot and VPC (see Supplementary Figures [Link], [Link]).

**Table 2:** Final parameter estimates (5th and 95th percentile)*

Fixed Effects (THETA)		*P*‐value†
**BIO**	1 (FIXED)	
**NN (number)**	25.0 (17.7‐35.1)	
**MTT (h)**		
**CHAPAS‐3**	0.82 (0.69‐0.96)	*P*<0.001 (dOFV = 21.4, df = 1)
**ARROW**	1.17 (1.02‐1.37)	
**Ka (1/h)**		
**CHAPAS‐3**	0.79 (0.37‐0.95)	*P*<0.001 (dOFV = 37.9, df = 1)
**ARROW**	1.27 (0.90‐1.62)	
**CL (l h^−1^)**		
**516GG|983TT**	6.94 (6.47‐7.61)	*P*<0.001 (dOFV = 154.7, df = 5)
**516GG|983TC**	3.93 (2.61‐5.65)	
**516GG|983CC**	0.74 (0.72‐0.75)	
**516GT|983TT**	4.90 (4.40‐5.46)	
**516GT|983TC**	1.36 (0.97‐1.76)	
**516TT|983TT**	1.92 (1.52‐2.33)	
**Vc (l)**	64.1 (49.1‐73.3)	
**Q (l h^−1^)**	17.1 (14.1‐20.9)	
**Vp (l)**	92.2 (80.1‐112.7)	

**Random Effects (ETA)** **		*P*‐value†
**BSVBIO**	42.2% (31.3%‐50.8%)	*P*<0.001 (dOFV = 19.7, df = 1)
**BOVBIO**	50.5% (42.3%‐55.9%)	*P*<0.001 (dOFV = 304.3, df = 1)
**BOVMTT**	78.0% (71.5%‐96.1%)	*P*<0.001 (dOFV = 443.3, df = 1)
**BOVKA**	57.7% (45.5%‐69.0%)	*P*<0.001 (dOFV = 96.7, df = 1)
**BSVCL**	36.9% (24.9%‐45.6%)	*P*<0.001 (dOFV = 64.2, df = 1)
**BOVCL**	26.6% (18.9%‐35.4%)	*P*<0.001 (dOFV = 26.9, df = 1)

**Error model (SIGMA)**		*P*‐value†
**Additive error (mg l^−1^)**	0.101 (0.067‐0.131)	*P*<0.001 (dOFV = 199.2, df = 1)
**Proportional error (%)**	6.72 (5.20‐7.90)	*P*<0.001 (dOFV = 678.5, df = 1)
**Increased error for sparse data**	2x (1.7x −2.5x)	*P*<0.001 (dOFV = 17.7, df = 1)

Final parameter estimates are typical population values estimated by the model. All clearance and volume parameters scaled allometrically to median weight of 15.4 kg.

* Estimated from nonparametric bootstrap (*n* = 200) of the final model.

** Expressed as approximate %CV on SD scale $\left(\sqrt{\text{ETA}}*100\right)$.

† Change in the objective function value after elimination of the parameter from the final model (dOFV > 10.83 corresponds to *P* < 0.001). BOVBIO, between occasion variability in bioavailability; BOVCL, between occasion variability in clearance; BOVMTT, between occasion variability in medium transit time; BOVKA, between occasion variability in absorption rate constant; BSVBIO, between subject variability in bioavailability; BSVCL, between subject variability in clearance; CL, clearance; BIO, bioavailability; Ka, absorption rate constant; MTT, mean transit time; NN, number of transit compartments; Q, inter‐compartmental clearance; Vc, volume of central compartment; Vp, volume of peripheral compartment.

The effect of body size on all clearance and volume parameters was accounted for using allometric scaling, which significantly improved model fit (18‐point drop in OFV) 43. No effect of age on the maturation of clearance could be detected. After adjusting for body size, the main predictor of clearance was the effect of *CYP2B6* genotype, categorized into six subgroups based on the combined effect of 516G>T and 983T>C SNP variants present in our population (Tables 2, 3). *CYP2B6* genotype explained 44.5% and 14.7% of BSV in clearance and oral bioavailability respectively. Exclusion of individuals with missing genotype did not have a significant effect on final results.

**Table 3:** PK exposures of different metabolic subgroups determined by 516GT|983TC SNP vector

**SNP vector**	**MET**	**Pts** *	**CL (l h** ^**−1**^ **)** *	**C12h (mg** **l** ^**−1**^ **)** **, †	**C12h < 1 (mg l** ^**−1**^ **)** **	**1 > C12h < 4 (mg l** ^**−1**^ **)** **	**C12h > 4 (mg l** ^**−1**^ **)** **	**C24h (mg** **l** ^**−1**^ **)** **, †	**AUC (mg h** ^**−1**^ **l** ^**−1**^ **)** **, †
**516GG|983TT**	EM	56 (33.1%)	6.94	1.55 (0.51–2.94)	40 (22%)	132 (74%)	6 (3%)	0.86 (0.26–2.02)	37.53 (14.26–75.12)
**516GG|983TC**	IM	10 (5.9%)	3.93	2.03 (1.19–4.53)	7 (16%)	28 (62%)	10 (22%)	1.33 (0.65–3.66)	46.30 (30.65–118.08)
**516GG|983CC**	USM	1 (0.6%)	0.74	18.22 (11.84–22.76)	0 (0%)	0 (0%)	6 (100%)	17.28 (11.20–21.63)	438.94 (286.10–548.20)
**516GT|983TT**	IM	59 (34.9%)	4.90	2.20 (0.97–4.40)	19 (10%)	132 (69%)	40 (21%)	1.54 (0.58–3.54)	56.05 (25.16–105.47)
**516GT|983TC**	SM	12 (7.1%)	1.36	7.79 (3.66–24.59)	0 (0%)	6 (17%)	29 (83%)	6.97 (3.24–23.07)	258.42 (64.81–548.77)
**516TT|983TT**	SM	31 (18.4%)	1.92	7.55 (2.40–14.74)	7 (6%)	20 (18%)	82 (75%)	6.61 (1.93–13.35)	175.98 (49.61–356.44)

Data are population median (5th–95th percentile) or number (percentage). Based on all patients from ARROW and CHAPAS‐3 trials (missing genotype estimated by mixture model).

* CL refers to typical population value estimated by the model for a patient with median weight of 15.4 kg (combined ARROW and CHAPAS‐3 data).

** CHAPAS‐3 data only (due to differences in dosing between studies, see Methods).

† Value for each PK visit estimated by the model, multiple measurements used to calculate geometric mean for every patient which were then used to calculate median and percentiles for each subgroup. MET, metabolic subgroup 32: EM (extensive metabolizers), 516GG|983TT; IM (intermediate metabolizers), 516GG|983TC or 516GT|983TT, SM (slow metabolizers), 516TT|983TT or 516GT|983TC; USM (ultra‐slow metabolizers), 516GG|983CC. The green shading indicates groups of patients who would be significantly overexposed if dose optimization were conducted based only on SNP 516GT.

The absorption rate constant (*k*_*a*_) and the absorption mean transit time (MTT) were 1.6‐fold larger and 1.4 times longer in ARROW compared with CHAPAS‐3. Splitting of the new double‐scored efavirenz tablets used in CHAPAS‐3 was not found to affect efavirenz bioavailability. No other covariate (see Methods) was found to significantly improve the model fit. We did not detect any systematic differences between the assays and labs employed in the analysis.

The model fit was markedly improved by inclusion of a correction parameter to allow for larger residual unexplained variability for all samples obtained after self‐recorded efavirenz intake. This includes all predose intensive PK samples and all the sparse PK samples from CHAPAS‐3. The residual variability for those samples was twice as large as in the rest of the data.

### Simulations

**Figure 1:**
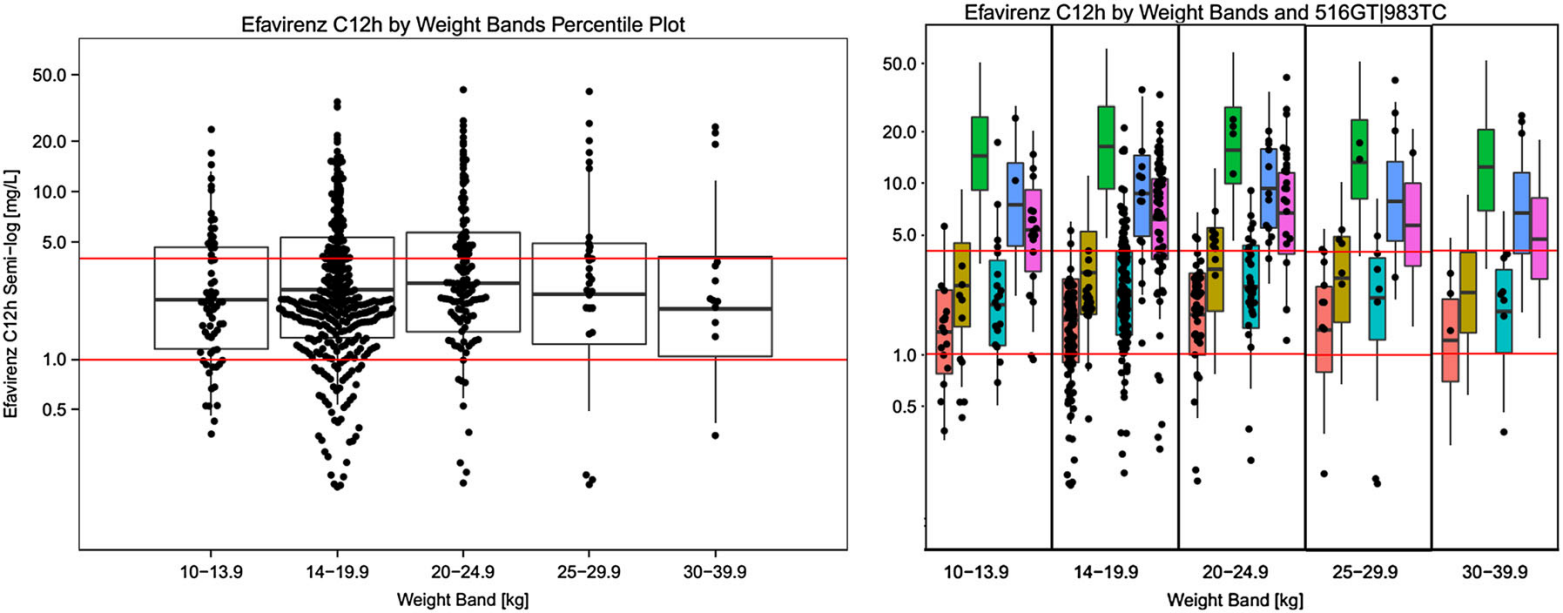
Individual mid‐dose concentrations estimated by the population pharmacokinetic model (black dots) plotted on top of the mid‐dose concentrations (percentile plots) simulated across weight‐bands (left) and by different *CYP2B6* 516GT|983TC subgroups (right). Red horizontal lines correspond to efavirenz concentrations of 1 mg l^−1^ and 4 mg l^−1^ 3. Breaks in the percentile plot correspond to 25th, median and 75th percentile and whiskers correspond to 5th and 95th percentile of the simulated data. CYP2B6 (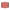) 516GG|983TT; (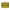) 516GG|983TC; (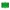) 516GG|983CC; (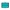) 516GT|983TT; (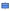) 516GT|983TC; (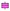) 516TT|983TT

Simulations were performed to predict exposures in African children based on their weight and genotype, when dosed according to the regimen used in CHAPAS‐3. Median mid‐dose concentrations were comparable across weight bands but noticeable differences were observed between the *CYP2B6* genotype subgroups (Figure 1, Table 3).

A dose optimization strategy for African children was devised by categorizing subjects into four phenotypic subgroups based on their composite genotype vector 516G>T|986T>C, similarly to Dooley *et al.* 32 (presented in Table 3). The proposed dose adjustment between metabolic subgroups is based on optimal ratios of 1:0.66:0.33:0.1 for EM:IM:SM:USM (extensive, intermediate, slow and ultra‐slow metabolizers), respectively, and is outlined in Table 4.

**Table 4:** Dosage tested in CHAPAS‐3 *vs*. proposed genotype adjusted dose optimization

**CHAPAS‐3**		**Based on 516GT|986TC**				
**Weight (kg)**	**Dose**	**Weight (kg)**	**EM**	**IM**	**SM**	**USM**
			**1**	**0.66**	**0.33**	**0.1**
**10–13.9**	200	**10–13.9**	300	200	100	50
**14–19.9**	300	**14–19.9**	400	300	150	50
**20–24.9**	400	**20–24.9**	600	400	200	100
**25–29.9**	400	**25–29.9**	600	400	200	100
**30–39.9**	400	**30–39.9**	600	400	200	100

EM (extensive metabolizers), 516GG|983TT; IM (intermediate metabolizers), 516GG|983TC or 516GT|983TT; SM (slow metabolizers), 516TT|983TT or 516GT|983TC; USM (ultra‐slow metabolizers), 516GG|983CC 32. The dose recommendations were rounded to the nearest full entity of currently available formulations (50 mg capsule, 100 mg capsule and 600 mg double scored tablets allowing doses of 200 mg, 300 mg, 400 mg and 600 mg).

**Figure 2:**
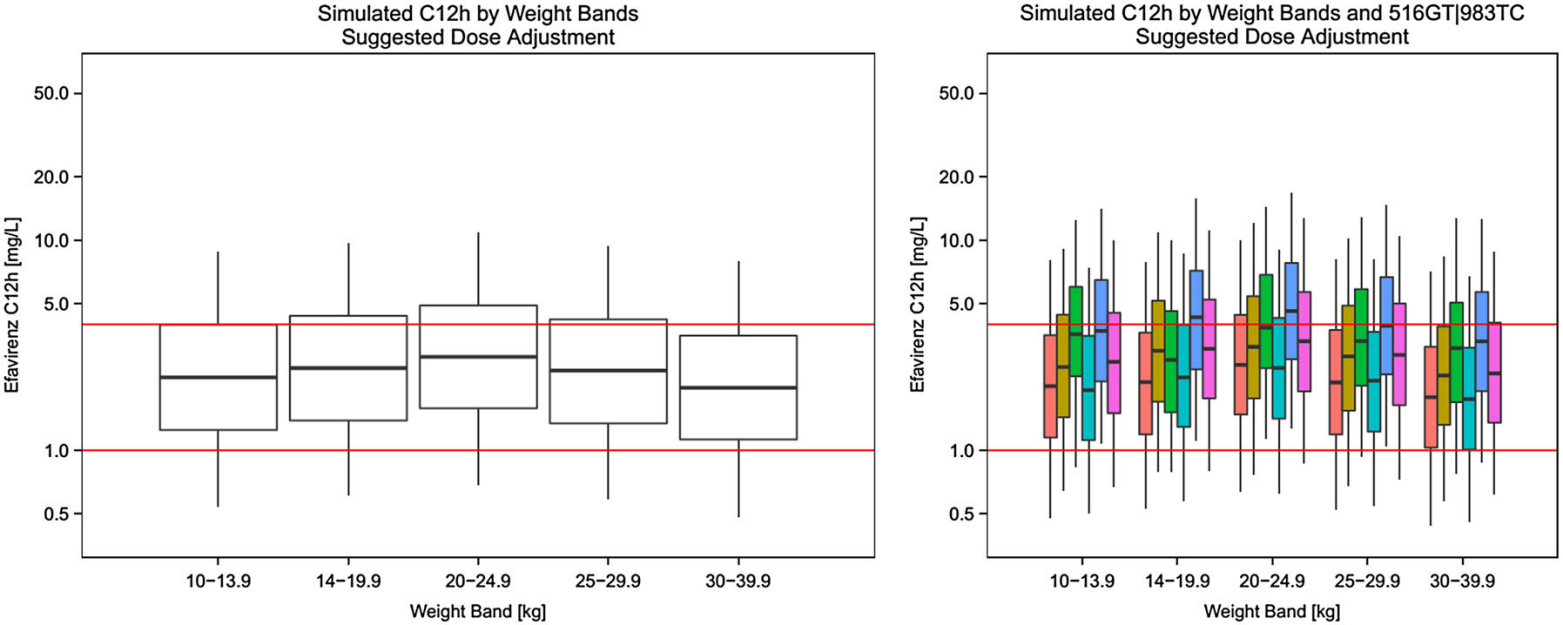
Simulated mid‐dose concentrations across weight‐bands (left) and by different 516GT|983TC genotypes (right) based on suggested most optimal dosing. Red horizontal lines correspond to efavirenz concentrations of 1 mg l^−1^ and 4 mg l^−1^ 3. Breaks in the percentile plot correspond to 25th, median and 75th percentile and whiskers correspond to 5th and 95th percentile of the simulated data. CYP2B6 (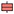) 516GG|983TT; (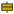) 516GG|983TC; (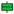) 516GG|983CC; (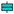) 516GT|983TT; (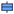) 516GT|983TC; (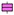) 516TT|983TT

The predicted exposures based on the suggested dose‐optimization algorithm are presented in Figure 2 and Supplementary Table S1. The suggested dosing approach ensured adequate drug exposure in all simulated weight bands (Figure 2, left panel), and reduced the differences due to metabolic status (Figure 2, right panel).

## Discussion

Efavirenz pharmacokinetics in Africans has previously been shown to be affected by the combined effect of SNPs 516G>T and 983T>C 8, 12, 14, 17, 32. The current investigation confirms those findings and is the first analysis to quantify the effect of the *CYP2B6* 516G>T|983T>C SNP vector on efavirenz clearance in African children using nonlinear mixed‐effects modelling. The use of modelling provides a tool to concomitantly account for multiple effects such as genotype and weight, and a platform to derive a dose adjustment strategy based on these effects.

Numerous studies in adults and children have reported a significant effect of *CYP2B6* 516G>T on efavirenz clearance. Our analysis shows that presence of one variant allele in 516G>T causes clearance to drop by 34%, while the reduction reaches 72% for homozygous mutants, which is in line with previously reported reductions of 20–47% and 58–80%, respectively 13, 20, 24, 26, 45, 46, 47. Our findings also show that the effect of SNP 516G>T is significantly modified by the 983T>C (i.e. in wild type 516G>T individuals presence of a single variant allele in 983T>C causes a 43% drop in clearance and 89% if no functional allele is present), confirming associations found by Holzinger *et al*. 8 and in a number of African studies 12, 14, 32. This polymorphism is virtually absent in individuals of European ancestry 8, 9 and was not detected in a study of Cambodian patients 24. The combined effect of the *CYP2B6* 516G>T|983T>C SNP vector on efavirenz clearance has previously been reported distinguishing four phenotypic subgroups 32. In our study, we were able to further characterize this effect using six *CYP2B6* 516G>T|983T>C SNP variants. Similarly to previous reports we show that, despite its low prevalence, SNP 983T>C is not only a significant predictor of efavirenz clearance, but it is responsible for a larger drop in metabolic rate than 516G>T (i.e. 29% drop in clearance in 516G>T heterozygote *vs*. 43% drop in 983T>C heterozygote, when no other polymorphisms were present) 8, 12. No further significant genetic associations were detected.

A genome‐wide association study by Holzinger *et al*. identified rs4803419 as another significant polymorphism in *CYP2B6* affecting efavirenz clearance 8. The effect of SNP rs4803419 becomes significant only for homozygous mutants who are wild type for 516G>T and 983T>C and no such variants were present in our population. Although this finding was recently replicated in South African patients, the investigators concluded this effect was negligible in comparison to 516G>T and 983T>C 12.

Results from the simulations (Figure 1) showed that, even though the dosage guidelines tested in CHAPAS‐3 result in average mid‐dose concentrations within target range of 1.0–4.0 mg l^−1^ 3, the effect of *CYP2B6* genotype leads to large differences within each weight band. In particular, children with slower *CYP2B6* genotypes (516GG|983CC, 516GT|983TC and 516TT|983TT) were overexposed, while the fastest metabolizers (516GG|983TT) achieved exposures at the bottom of the therapeutic range. Over 20% of children in the study with the 516GG|983TT genotype had efavirenz concentrations below the proposed minimum target concentration of 1.0 mg l^−1^ (Table 3). Moreover, our model indicates that disregarding the effect of the 983T>C SNP and basing dose optimization only on 516G>T could lead to exposures significantly higher than the therapeutic range 3 in ~14% of African patients with 983TC or 983CC genotypes (Table 3 in green). This suggests that genotype‐based dose optimization in African children should take into account both 516G>T and 983T>C SNPs.

**Figure 3:**
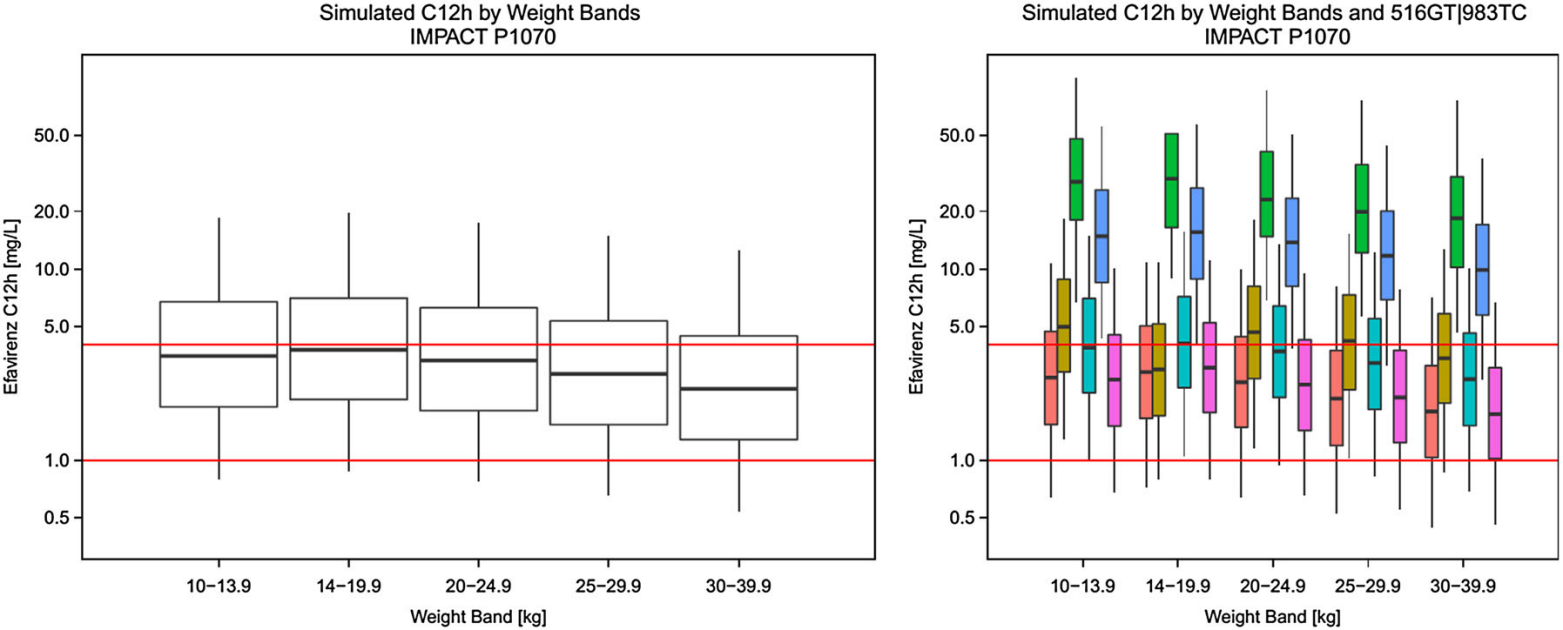
Simulated mid‐dose concentrations across weight‐bands (left) and by different 516GT|983TC genotypes (right) based on dose recommendations tested in IMPACT study P1070 applied to our population. Red horizontal lines correspond to efavirenz concentrations of 1 mg l^−1^ and 4 mg l^−1^ 3. Breaks in the percentile plot correspond to 25th, median and 75th percentile and whiskers correspond to 5th and 95th percentile of the simulated data. CYP2B6 (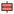) 516GG|983TT; (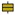) 516GG|983TC; (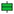) 516GG|983CC; (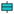) 516GT|983TT; (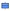) 516GT|983TC; (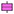) 516TT|983TT

The only available guidelines on genotype‐adjusted paediatric dosage were recently formulated for patients under 3 years of age by the Panel on Antiretroviral Guidelines for Adults and Adolescents and are currently being tested in the IMPAACT study P1070 10, 11. The dose‐adjusted strategy was developed based on results of an analysis by Salem *et al*. 23 and preliminary results of IMPAACT P1070 48 and proposed different dosing for individuals with 516GG or 516GT genotype versus 516TT. In contrast to previous paediatric studies 46, 49, Salem *et al*. did not detect significant differences in clearance rate between patients with 516GG and 516GT genotypes, and the effect of 983T>C was not evaluated. Their findings might differ from the current investigation for several reasons: the study by Salem *et al.* included a smaller number of patients, the lowest age of participants was only 2 months, the tested population comprised patients of various races and the formulations included capsules and liquid. According to simulations from the current model, direct application of that strategy in African children could result in exposures above the therapeutic range 3 in a large proportion of patients who are either heterozygous for 516G>T or wild type with 983TC or 983CC genotypes (Figure 3).

Our recommendations were simplified to the previously described four metabolic subgroups determined by the composite genotype vector 516G>T|983T>C outlined in Table 3 32. The clearance between EM:IM:SM:USM drops as follows: 1:0.6:0.24:0.1, which is remarkably similar to the ratios detected by Dooley *et al*. in African adults (1:0.6:0.26:0.08, respectively) 32. Our dosage algorithm presented in Table 4 (1:0.66:0.33:0.1) was adjusted to maximize the use of currently available solid formulations. A similar dose adjustment pattern (1:0.66:0.33) was previously successfully implemented based on phenotypic differences in an adult study by Mello *et al.* 50.

The results of the few dose reduction studies guided by *CYP2B6* genotype conducted in developed countries highlighted improved treatment tolerability and cost‐effectiveness in adults 50, 51, 52, 53. These results were confirmed by recent cost‐effectiveness analysis of this practice in American adults 54. It could be speculated that in resource‐limited settings, cost and logistical challenges would make implementation of such practice difficult. Nonetheless, decreasing price and broader availability of genotyping technology and economic development open future scenarios in which genotype‐based dosing approaches may be economically viable and beneficial even in developing countries. The IMPAACT P1070 study, which is currently being conducted in HIV‐infected infants and children, should give more insight into the practical implications of genotyping in low‐ and middle‐income countries.

Similarly to previous paediatric studies, the average clearance value (before inclusion of genotype effect) was higher than findings in adults: 14.34 L h^−1^ *vs*. 7.5 to 11.7 L h^−1^ (both after scaling with allometry up to 70 kg) 5, 21, 24, 45, 46. This is consistent with reports that clearance in children of age 1–4 years exceeds adult values 27, 46, 55. In keeping with previously published paediatric models, the effect of size on clearance and volume was explained through allometric scaling 23, 43, 46. Unlike Salem *et al*., we did not observe age‐related maturation of clearance, but the previous analysis showed that 90% of maturation was reached by the age of 9 months 23 and the majority of patients in the current study were >3 years.

We detected significant differences in absorption parameters between CHAPAS‐3 and ARROW, possibly related to the use of different formulations (tablets in the former and mostly capsules in the latter). The formulations were assumed to be bioequivalent and indeed no formulation effect on bioavailability was detected.

Due to the availability of data from multiple sampling occasions within the same patient, it was possible to characterize both BSV and BOV in the PK parameters. Large BOV was identified for absorption parameters and bioavailability. Drug absorption is widely known to be a variable phenomenon, depending on occasion‐specific factors, such as food intake, gastric emptying times and GI tract pH. In the current study, other factors may have contributed to inflating BOV (in particular bioavailability), including differences between actual and self‐reported intake times for the sparse data, lack of accurate intake history before the last dose, lack of information on accompanying food consumption. The fact that the information about intensively sampled occasions was more accurate was accounted for with the introduction of a scaling factor on residual unexplained variability (2‐fold larger for sparse data).

The current study had several limitations. As mentioned, the dosage timing and food co‐administration was not recorded beyond the last intake. Previous studies showed that efavirenz PK was affected by adherence and food effects 2, 21, 25. Polymorphisms in accessory pathways including *CYP2A6* and *CYP3A4* or *UGT* were not assessed and the effect of *CYP2B6* 785A>G was only evaluated in patients from the ARROW study. Despite several reports suggesting that efavirenz metabolism is affected by polymorphisms in those pathways, the genome‐wide association study by Holzinger *et al.* showed that their effect was significantly less dramatic than for 516G>T and 983T>C 8.

Furthermore, no PK/PD relationship was explored in this analysis for either efficacy or toxicity, but currently accepted therapeutic ranges were used as cut‐offs guiding dose optimization. These targets were generated in an adult cohort and have recently been brought into question, suggesting that lower efavirenz concentrations might be sufficient to provide viral suppression 17; however, no alternative has been suggested to date.

Lastly, our study was underpowered to determine the effect of tuberculosis treatment on efavirenz concentrations, but recent findings suggest that the inducing effect of rifampicin on clearance is counterbalanced by a concentration‐dependent inhibitory effect of isoniazid that could explain contradictory conclusions from previous studies 7, 24, 32.

## Conclusions

Our study suggests that genotype‐adjusted efavirenz dosage in African children should be based on the composite 516G>T|983T>C SNP vector, due to significant modification of clearance rates caused by SNP 983T>C genotype, whose prevalence in Africans is much higher than in other populations. Using nonlinear mixed‐effects modelling, we quantified this effect and suggest that a dose optimization algorithm 1:0.66:0.33:0.1 (EM:IM:SM:USM, respectively) would provide more balanced drug exposures between individuals with a different metabolic status while maximizing the potential of using the new double‐scored efavirenz tablets tested in the CHAPAS‐3 study. The findings warrant further studies evaluating the genotype‐based dosing approach and the feasibility of genotyping in resource‐limited settings.

## Competing Interests

All authors have completed the Unified Competing Interest form and declare: AB, AC, VM, CK, AD, ASM, DMG, HM and DB received support through grants from European Developing Countries Clinical Trials Partnership (EDCTP); AC, AD, ASM and DMG additionally received grants from Medical Research Council (MRC) UK; HM additionally declares support in part by the National Research Foundation of South Africa, grant 90729; AO received support in form of grants from Janssen, ViiV and Tandem Nano, as well as personal fees from Merck was issued a patent “Compositions of efavirenz”. No other support from any organization for the submitted work; no financial relationships with any organizations that might have an interest in the submitted work in the previous 3 years and no other relationships or activities that could appear to have influenced the submitted work are to be declared for any of the authors.

## Funding statement

ARROW is funded by the MRC UK and the UK Department for International Development. ViiV Healthcare/GlaxoSmithKline donated first‐line drugs for ARROW and provided funding for viral load assays.

CHAPAS‐3 was funded by EDCTP (IP.2007.33011.006), MRC UK, Department for International Development UK, Ministerio de Sanidad y Consumo Spain. Cipla Ltd donated first‐line antiretrovirals.

The drug assays were supported in part by the National Institute of Allergy and Infectious Diseases of the National Institutes of Health (UM1 AI068634, UM1 AI068636 and UM1 AI106701, U01 AI068632), the Eunice Kennedy Shriver National Institute of Child Health and Human Development (NICHD), and the National Institute of Mental Health (AI068632).

We thank all the patients and the staff from all he centres involved in the ARROW and CHAPAS‐3 studies. The studies were a joint collaboration of the following institutions: JCRC, Kampala, Uganda; MRC/UVRI Uganda Research Unit on AIDS, Entebbe, Uganda; Baylor, Uganda; Paediatric Infectious Disease Centre, Mulago Hospital, Uganda; University of Zimbabwe, Harare, Zimbabwe; University Teaching Hospital, School of Medicine, Lusaka, Zambia; JCRC, Gulu, Uganda; MRC CTU, London, UK; Radboud University Nijmegen Medical Centre, Nijmegen, the Netherlands; University of Cape Town, Cape Town, South Africa.

*The Division of Clinical Pharmacology at the University of Cape Town would like to gratefully acknowledge Novartis Pharma for their support of the development of pharmacometrics skills in Africa*.

*The content is solely the responsibility of the authors and does not necessarily represent the official views of any funders*.

## Supporting information


**Table S1** Simulated values of mid‐dose concentrations obtained after suggested dose optimization and proportions of patients of less than 1 mg l^−1^, between 1 mg l^−1^ and 4 mg l^−1^, and more than 4 mg l^−1^
**Figure S1** Goodness of fit plots. Top left, observations *vs*. population predictions; top right, observations *vs*. individual predictions; bottom left, conditional weighted residuals *vs*. time after dose; bottom right, absolute values of individual weighted residuals *vs*. individual predictions
**Figure S2** Visual predictive check of the final model by 516GT|983TC SNP vector in semi‐log scale. Hollow points, observations; red solid line, median of observed data; red line with breaks, 5th and 95th percentile of observed data; orange fill area, 95% CI of simulated median; blue fill area, 95% CI of simulated 5th and 95th percentile

Supporting info item

Supporting info item

Supporting info item
