# Supplementary material for: The impact of genetic polymorphisms on the pharmacokinetics of efavirenz in African children
Source: Br J Clin Pharmacol. 2016 Apr 25;82(1):185–98. doi: 10.1111/bcp.12934 (PMC4917805; doi:10.1111/bcp.12934)
Supplement: Supplementary file 1 — Supporting info item [file BCP-82-185-s001.docx]

| **Table 1S Simulated values of mid-dose concentrations obtained after suggested dose optimisation and proportions of patients <1mg/L, 1mg/L>&<4mg/L and >4mg/L** | | | | | | | |
| --- | --- | --- | --- | --- | --- | --- | --- |
| **Weight Band**  **[kg]** | **Metabolic group** | **FM** | **IM** | | **SM** | | **USM** |
|  | **SNP Vector** | **516GG\| 983TT** | **516GG\| 983TC** | **516GT\| 983TT** | **516GT\| 983TC** | **516TT\| 983TT** | **516GG\| 983CC** |
| **10-13.9** | **Dose [mg]** | 300 | 200 | | 100 | | 50 |
|  | **C12h [mg/L]** | 2.02  (0.47-8.04) | 2.49  (0.64-9.11) | 1.93  (0.50-7.42) | 3.71  (1.08-14.01) | 2.63  (0.67-10.04) | 3.57  (0.83-12.43) |
|  | **>4 [mg/L]** | 21% | 29% | 20% | 46% | 30% | 45% |
|  | **1> & <4 [mg/L]** | 60% | 58% | 59% | 50% | 57% | 49% |
|  | **<1 [mg/L]** | 20% | 13% | 21% | 4% | 12% | 7% |
| **14-19.9** | **Dose [mg]** | 400 | 300 | | 150 | | 50 |
|  | **C12h [mg/L]** | 2.11  (0.52-7.86) | 2.98  (0.79-10.90) | 2.22  (0.57-8.68) | 4.30  (1.10-15.77) | 3.04  (0.79-11.12) | 2.70  (0.79-9.99) |
|  | **>4 [mg/L]** | 22% | 36% | 24% | 53% | 37% | 30% |
|  | **1> & <4 [mg/L]** | 60% | 55% | 59% | 43% | 54% | 60% |
|  | **<1 [mg/L]** | 19% | 9% | 16% | 4% | 9% | 10% |
| **20-24.9** | **Dose [mg]** | 600 | 400 | | 200 | | 100 |
|  | **C12h [mg/L]** | 2.55  (0.63-9.98) | 3.11  (0.76-12.07) | 2.47  (0.62-8.98) | 4.6  (1.27-16.79) | 3.31  (0.87-12.68) | 3.84  (1.13-14.30) |
|  | **>4 [mg/L]** | 29% | 38% | 28% | 57% | 41% | 47% |
|  | **1> & <4 [mg/L]** | 58% | 54% | 58% | 40% | 52% | 48% |
|  | **<1 [mg/L]** | 13% | 8% | 14% | 3% | 7% | 4% |
| **25-29.9** | **Dose [mg]** | 600 | 400 | | 200 | | 100 |
|  | **C12h [mg/L]** | 2.10  (0.52-8.12) | 2.80  (0.67-10.20) | 2.14  (0.54-8.14) | 3.92  (1.04-14.74) | 2.84  (0.73-10.42) | 3.31  (0.93-12.77) |
|  | **>4 [mg/L]** | 22% | 34% | 22% | 49% | 34% | 42% |
|  | **1> & <4 [mg/L]** | 58% | 55% | 60% | 47% | 56% | 53% |
|  | **<1 [mg/L]** | 19% | 12% | 18% | 5% | 10% | 6% |
| **30-39.9** | **Dose [mg]** | 600 | 400 | | 200 | | 100 |
|  | **C12h [mg/L]** | 1.80  (0.43-7.11) | 2.27  (0.57-8.42) | 1.76  (0.45-6.75) | 3.30  (0.87-12.59) | 2.32  (0.61-8.87) | 3.06  (0.77-12.74) |
|  | **>4 [mg/L]** | 16% | 24% | 16% | 40% | 26% | 36% |
|  | **1> & <4 [mg/L]** | 60% | 60% | 60% | 53% | 59% | 55% |
|  | **<1 [mg/L]** | 24% | 16% | 25% | 7% | 15% | 9% |

Data presented as median (5^th^-95^th^ percentile) or percentage. EM (extensive metabolisers) - 516GG|983TT; IM (intermediate metabolisers) - 516GG|983TC or 516GT|983TT, SM (slow metabolisers) - 516TT|983TT or 516GT|983TC; USM (ultra-slow metabolisers) - 516GG|983CC.
