# Supplementary figures and images for: The impact of genetic polymorphisms on the pharmacokinetics of efavirenz in African children
Source: Br J Clin Pharmacol. 2016 Apr 25;82(1):185–98. doi: 10.1111/bcp.12934 (PMC4917805; doi:10.1111/bcp.12934)

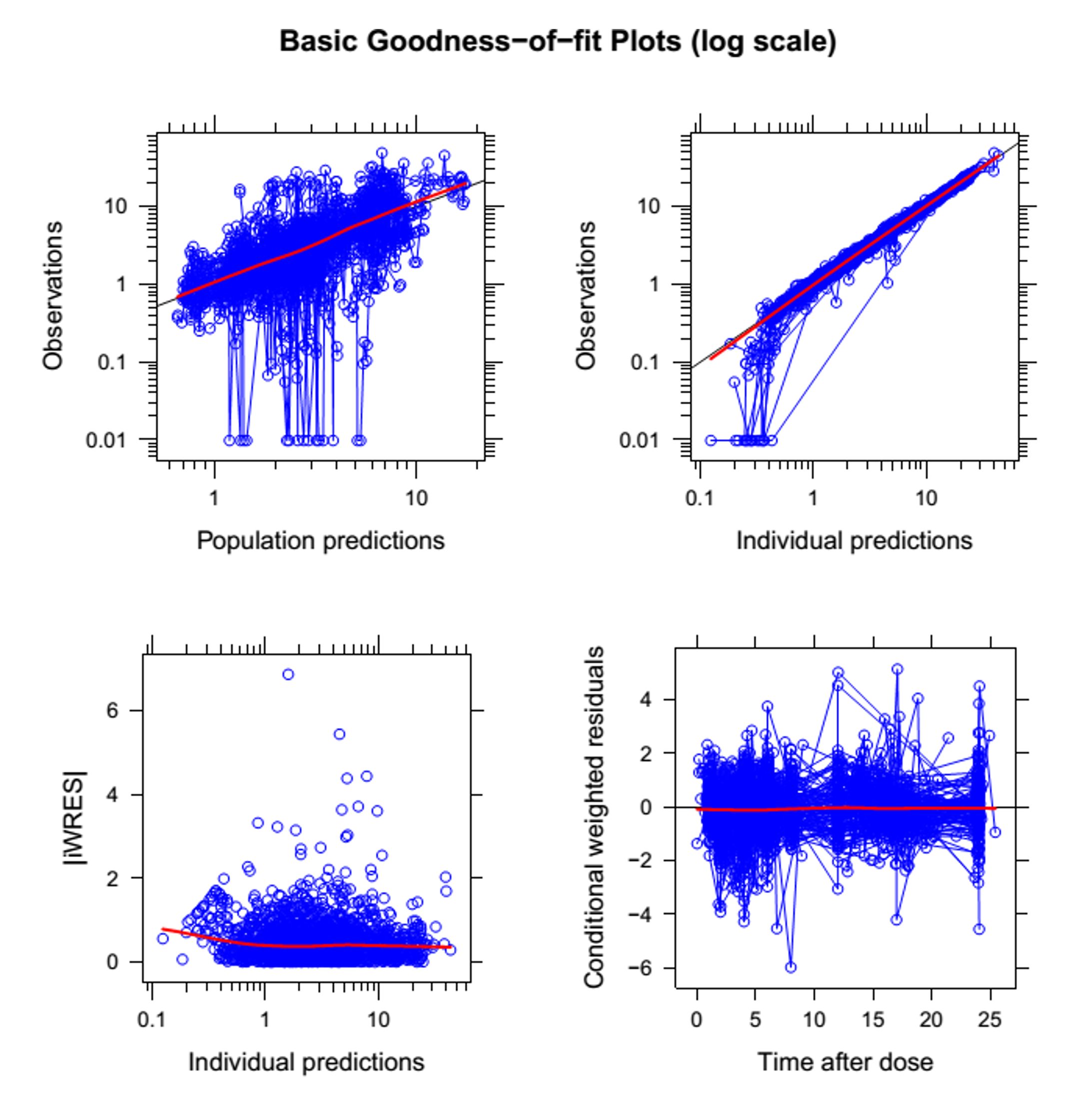

Supplement: Supplementary file 2 — Supporting info item [file BCP-82-185-s002.tif]

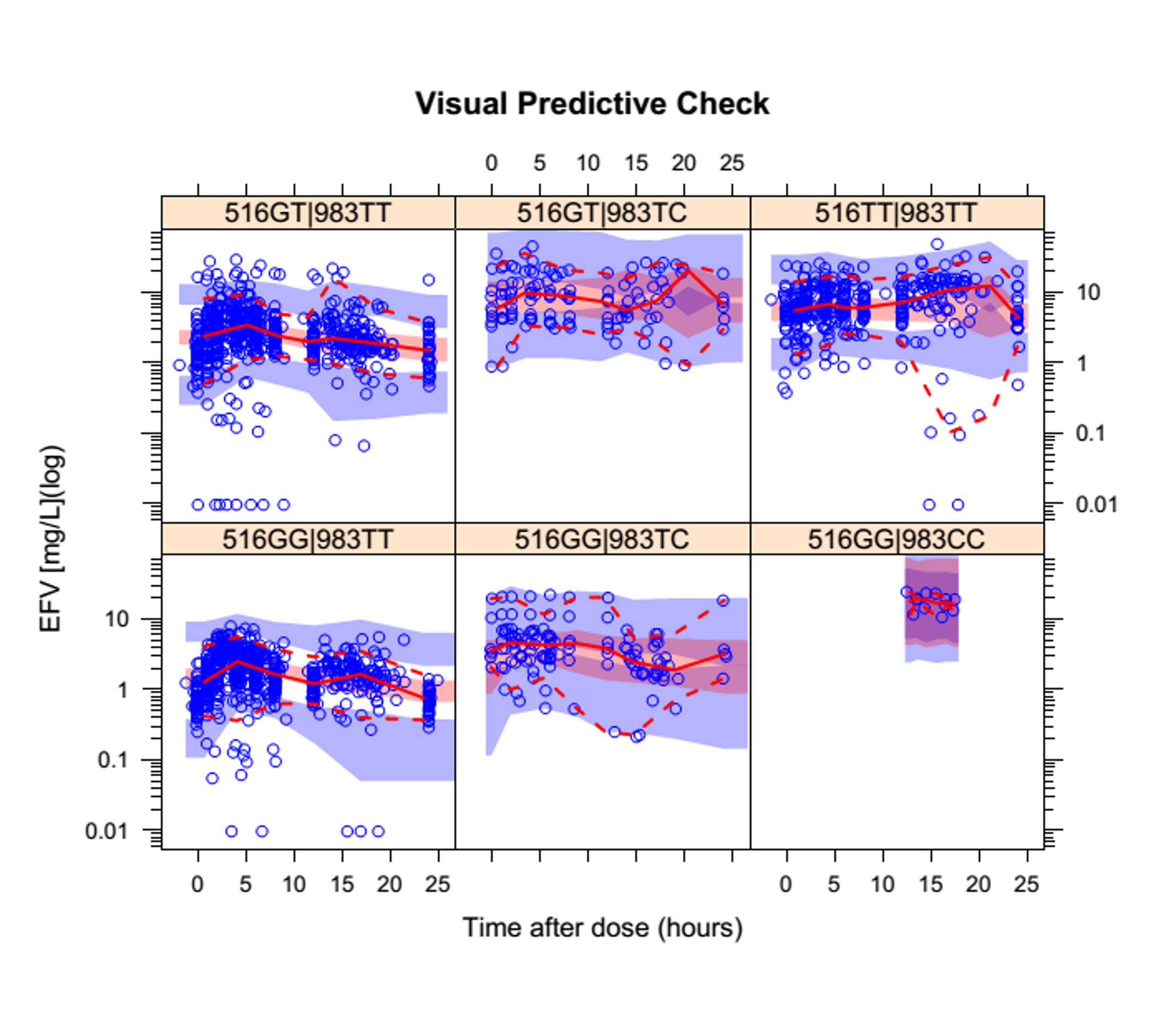

Supplement: Supplementary file 3 — Supporting info item [file BCP-82-185-s003.tif]
